# Nitrite accumulation and anammox bacterial niche partitioning in Arctic Mid-Ocean Ridge sediments

**DOI:** 10.1038/s43705-023-00230-y

**Published:** 2023-03-29

**Authors:** Rui Zhao, Andrew R. Babbin, Desiree L. Roerdink, Ingunn H. Thorseth, Steffen L. Jørgensen

**Affiliations:** 1 Department of Earth, Atmospheric and Planetary Sciences, Massachusetts Institute of Technology, Cambridge, MA 02139 USA; 2 Centre for Deep Sea Research, Department of Earth Science, University of Bergen, Bergen, 5007 Norway

**Keywords:** Water microbiology, Biogeochemistry

## Abstract

By consuming ammonium and nitrite, anammox bacteria form an important functional guild in nitrogen cycling in many environments, including marine sediments. However, their distribution and impact on the important substrate nitrite has not been well characterized. Here we combined biogeochemical, microbiological, and genomic approaches to study anammox bacteria and other nitrogen cycling groups in two sediment cores retrieved from the Arctic Mid-Ocean Ridge (AMOR). We observed nitrite accumulation in these cores, a phenomenon also recorded at 28 other marine sediment sites and in analogous aquatic environments. The nitrite maximum coincides with reduced abundance of anammox bacteria. Anammox bacterial abundances were at least one order of magnitude higher than those of nitrite reducers and the anammox abundance maxima were detected in the layers above and below the nitrite maximum. Nitrite accumulation in the two AMOR cores co-occurs with a niche partitioning between two anammox bacterial families (*Candidatus* Bathyanammoxibiaceae and *Candidatus* Scalinduaceae), likely dependent on ammonium availability. Through reconstructing and comparing the dominant anammox genomes (*Ca*. Bathyanammoxibius amoris and *Ca*. Scalindua sediminis), we revealed that *Ca*. B. amoris has fewer high-affinity ammonium transporters than *Ca*. S. sediminis and lacks the capacity to access alternative substrates and/or energy sources such as urea and cyanate. These features may restrict *Ca*. Bathyanammoxibiaceae to conditions of higher ammonium concentrations. These findings improve our understanding about nitrogen cycling in marine sediments by revealing coincident nitrite accumulation and niche partitioning of anammox bacteria.

## Introduction

The cycling of nitrogen in ecosystems is intricately controlled by a network of processes mediated by microorganisms. In an ecosystem, new bioavailable (or fixed) nitrogen is generated by diazotrophy, and can be converted back to N_2_ by two nitrogen loss processes: denitrification and anaerobic ammonium oxidation (anammox) [see the review in e.g. [1]]. The latter two anaerobic metabolisms are generally favored in low-oxygen environments, either the ocean’s pelagic oxygen minimum zones or benthic sediments [2]. Prior estimates suggest fixed nitrogen loss in the benthos is 1.3–3 times greater in magnitude than the water column on a global basis [3–5]. Therefore, sedimentary nitrogen loss processes play a crucial role in regulating the abundance of bioavailable nitrogen across marine habitats. Nitrite is a crucial substrate of both anammox and denitrification [6, 7], the availability of which exerts a profound control on the magnitude of nitrogen loss [8]. However, nitrite rarely accumulates to as high a level as nitrate and ammonium in marine sediments, leading the presence and transformation pathways of nitrite in this vast environment to be largely overlooked. Anammox bacteria are among the major consumers of nitrite, owing to their strict requirement of this compound to oxidize ammonium.

Since its discovery in the marine environment two decades ago [8], anammox has been shown to be a significant contributor to fixed nitrogen loss [e.g., [9]. Among the previously recognized marine anammox bacteria (affiliated with the families *Candidatus* Brocadiaceae and *Candidatus* Scalinduaceae), members of *Ca*. Scalinduaceae have been consistently detected in marine sediments [10–13], and several enrichment cultures have been obtained from coastal sediments [e.g., *Ca*. Scalindua japonica [14] and *Ca*. Scalindua profunda [15]]. However, while seemingly ubiquitous, *Ca*. Scalinduaceae may not be the only anammox bacterial family present in marine sediments. Recently, by examining metagenome-assembled genomes from Arctic Mid-Ocean Ridge (AMOR) sedimentss and from the groundwater environment, a new family of anammox bacteria was discovered (i.e., *Candidatus* Bathyanammoxibiaceae [16]). In the AMOR cores, both *Ca*. Scalinduaceae and *Ca*. Bathyanammoxibiaceae are confined within the nitrate-ammonium transition zone and *Ca*. Bathyanammoxibiaceae can sometimes significantly outnumber their counterparts of *Ca*. Scalinduaceae [16]. The co-existence of two functionally (almost) identical lineages in AMOR sediments raised the questions of whether these families occupy the same niche and what influence they might have on the distribution and transformations of nitrite.

Given their prevalence in deep-sea sediments, anammox bacteria have been suggested to play an important role in consuming the upward diffusive flux of ammonium and preventing the transport of ammonium from sediments to the overlying seawater [13]. Because nitrite is a necessary substrate for anammox [6], we hypothesize that, by analogy, the abundance and metabolic activity of anammox may also exert a strong influence on the distribution of nitrite in addition to ammonium. To test this hypothesis, we combined biogeochemical, microbiological, and genomic approaches to study the relationships between the distribution of dissolved nitrogen species and anammox bacteria and other nitrogen cycling groups. We first identified a phenomenon of nitrite accumulation in the nitrate-depletion zone in diverse marine sediment systems: the continental slope, mid-ocean ridges, and also hadal trenches. Through high-resolution analyses of microbial communities in two AMOR sediment cores with apparent nitrite accumulation, we observed niche partitioning of anammox bacteria between the families *Ca*. Scalinduaceae and *Ca*. Bathyanammoxibiaceae that are prevalent in marine sediments. Based on the newly generated high quality anammox genomes, we also proposed the likely underlying genetic mechanisms driving the observed niche partitioning.

## Results and discussion

### General geochemical context of GS14-GC04

The measurement of nitrite in the sediment porewater, along with ammonium and nitrate, was attempted for over a dozen sediment cores retrieved from the seafloor during our cruises to the Arctic Mid-Ocean Ridge (AMOR) area (e.g., [13, 17]). However, coherent nitrite profiles (defined as >2 consecutive depths with detectable nitrite concentrations) were only detected in two cores: GS14-GC04 and GS16-GC04 (see results below). These two cores offered an opportunity to explore the underlying mechanism(s) of nitrite accumulation, a unique geochemical phenomenon that has been well studied in seawaters of oxygen deficient zones [e.g., [18]] but not in marine sediments. Because the general geochemical context [13], microbiology data [13], and anammox bacteria communities [16] of core GS16-GC04 have been published previously, below we provide thorough descriptions for core GS14-GC04.

**Fig. 1:**
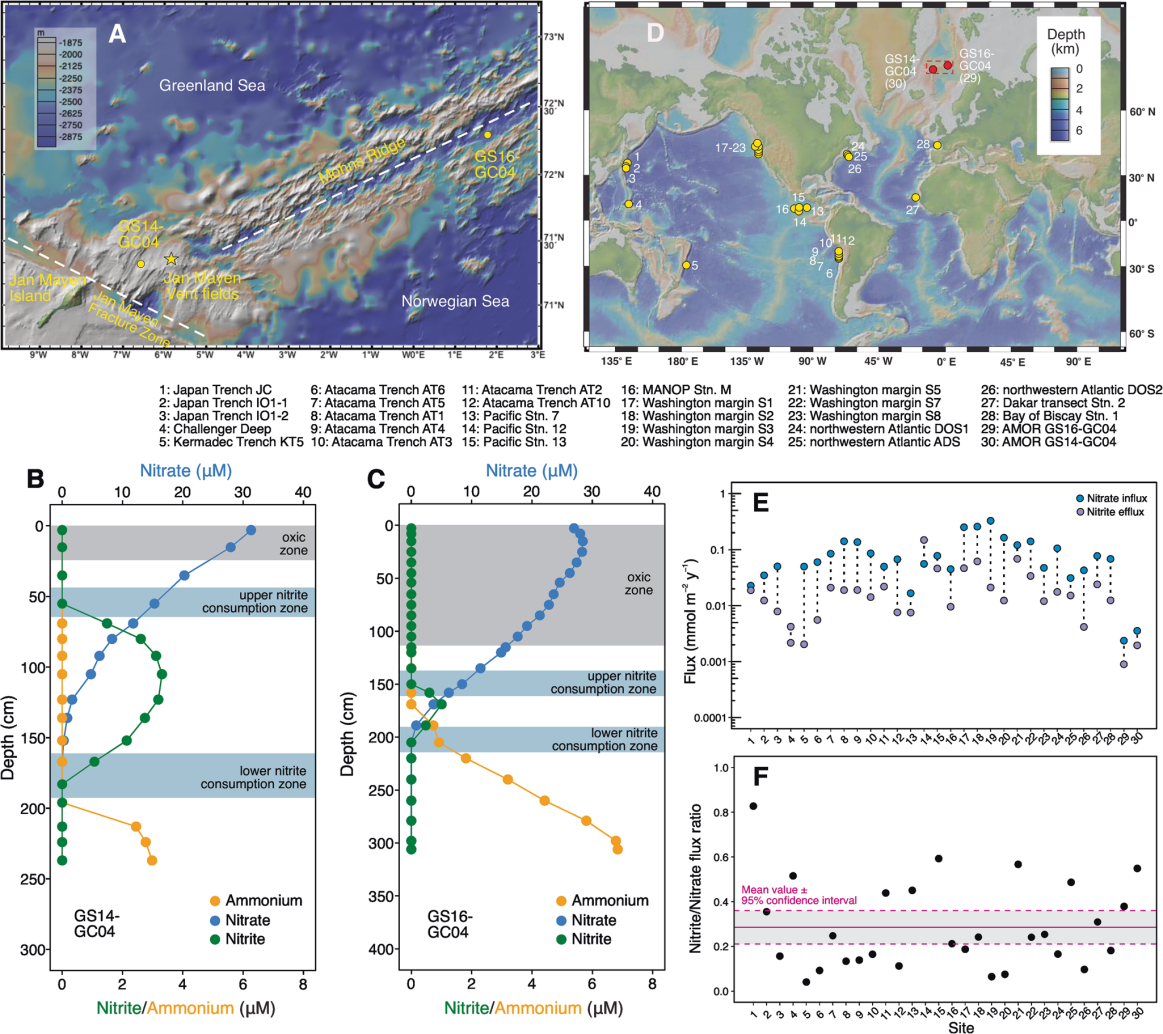
The occurrence of nitrite accumulation in sediment porewater of the Arctic Mid-Ocean Ridge and other locations. **A** Bathymetric map showing two coring sites (GS14-GC04 investigated in this study and GS16-GC04 investigated in ref. [13]) in the Arctic Mid-Ocean Ridge area where nitrite accumulation was observed. Also highlighted are the Jan Mayen Fracture Zone and the Mohns Ridge, as well as the Jan Mayen hydrothermal vent field (yellow star). Accumulation of nitrite in the two AMOR sediment cores. Porewater profiles of nitrate, nitrite, and ammonium of (**B**) GS14-GC04 and (**C**) GS16-GC04 are shown. The oxic zones and two (upper and lower) net nitrite consumption zones are highlighted by horizontal bands. **D** Sediment locations where nitrite accumulation in the sediment porewater was detected. The two AMOR sites are shown in red circles, while other sites are shown in yellow circles (See Fig. S3 for the porewater nitrite and nitrate profiles of individual sites). Maps in (**A**) and (**D**) were generated with GeoMapApp v. 3.6.14 (www.geomapapp.org), using the default Global Multi-Resolution Topography Synthesis basemap. (**E**) Nitrate influx and (combined upward and downward) nitrite efflux in the nitrate-depletion zones of the 30 sediment sites shown in (**D**). The paired fluxes for each site are connected with a black dotted line. **F** Calculated nitrite/nitrate flux ratio for the individual sites. The horizontal line denotes the mean value of the 30 sites, while the dashed lines represent the 95% confidence interval.

GS14-GC04 is a 2.4-m-long core retrieved from a 1050-m deep seamount 50 km west of the Jan Mayen hydrothermal vent fields (Fig. 1A) on the Arctic Mid-Ocean Ridge where white smoker hydrothermal vents were reported [19, 20]. Total organic nitrogen content (Fig. S1A) in the retrieved sediments of GS14-GC04 was measured to be in the range of 0.06–0.11%, while the total organic carbon content was measured to be less than 0.5 wt% (Fig. S2A). Thus, the calculated carbon to nitrogen ratio (C/N) fell generally in the range of 2–4 (Fig. S1B). Oxygen was measured to be only 15 µM at the top of the recovered core and was depleted within 23 cm below the seafloor. Below the depletion depth of oxygen, dissolved Mn accumulated in the porewater (Fig. S2B), a phenomenon also present in other sediment cores retrieved from the AMOR region [13]. Porewater pH fell between 7.6 and 7.8 (Fig. S1C), similar to those in other AMOR cores [13]. Dissolved Fe was not detected throughout the core (Fig. S1D), indicating that the reduction of Fe is not important in the recovered sediments. GS14-GC04 exhibited higher concentrations of dissolved inorganic carbon (DIC) (Fig. S2) than GS16-GC04 and the other AMOR cores without significant hydrothermal influences [13], indicating higher organic matter degradation activity in GS14-GC04. Despite the uppermost sediments of GS14-GC04 potentially been lost during coring (see Supplementary Note 1), the oxygen penetration depth of this core was shallower than the non-hydrothermal sites (e.g., ~110 cm in GS16-GC04 (Fig. 1C and S2D) and 35–100 cm in the other three cores previously described in [13]) and does not affect our interpretation of deeper anaerobic microbes and their metabolisms.

### Accumulation of nitrite in the nitrate-depletion zone

In contrast to GS16-GC04 (Fig. 1C) and the other AMOR cores previously described in [13] where the counter gradients of nitrate and ammonium converge within the thin nitrate-ammonium transition zone, core GS14-GC04 features a vertical separation between the downward flux of nitrate and the upward flux of ammonium. Nitrate in GS14-GC04 decreased with depth and was depleted around 130 cm (Fig. 1B). Yet, ammonium in this core was not detected in the porewater until 213 cm, well below the nitrate-depletion depth (Fig. 1B).

Unlike most AMOR sediment cores where nitrite was routinely measured but generally undetectable throughout all measured depths [17], nitrite in GS14-GC04 accumulated around the nitrate-depletion zone (50–180 cm), with a concentration maximum (~3 µM) at 105 cm (Fig. 1B). A similar nitrite accumulation, albeit of lower magnitude (~1 µM) and shorted vertical span (150–200 cm), was also detected in the nitrate-depletion zone of GS16-GC04 (Fig. 1C). By searching published literature, we found that such accumulation of nitrite around the nitrate-depletion zone can be seen in 28 additional globally distributed sediment cores (Fig. 1D; See the detailed nitrite, nitrate, and ammonium profiles in individual cores in Supplementary Fig. S3). Such accumulation was mainly detected in sediments on the continental slopes [e.g., [21–24]], along the mid-ocean ridges [25] of the Pacific and Atlantic Oceans, and within hadal trenches in the Pacific [10, 26, 27], rather than along the continental margin or in the abyssal plains (Fig. 1D). Most of these sites accmulte nitrite within the nitrate-ammonoum transiztion zone (Fig. S3), where the anammox reaction occurs [13]. This alignment suggests a potential link between anammox bacteria and the observed nitrite accumulation. Nitrite accumulation was hardly detected in the upper few meters of sediments (i) of continental margins because nitrate penetration is too shallow to be properly resolved without dedicated microscale measurements, and (ii) of abyssal plains [e.g., [26]] because high porewater concentrations of nitrate and O_2_ are present deep into the sediments [26, 28–30]. Through this comparison, it is likely that the observed nitrite accumulation in sediments of continental slopes, mid-ocean ridges, and hadal trenches is tightly associated with low concentrations of nitrate within the nitrate-depletion zone, which in turn is caused by moderate levels of organic matter flux. Although our compilation suggests that nitrite accumulation is distributed globally at sediment sites of intermediate organic carbon rain, more systematic sampling is needed to assess the frequency and mechanistic controls on nitrite accumulation in marine sediment systems.

While not generally reported in marine sediments, nitrite accumulation coincident with declining nitrate concentrations is often observed in other stratified aquatic environments like the water columns of the Black Sea [31, 32] and Golfo Dulce [33], the freshwater Lake Tanganyika [34], hypersaline Lakes Vanda and Bonney [35] in the McMurdo Dry Valleys in Antarctica, river and estuary sediments [36, 37], subtropical mangrove sediments [38], and denitrifying biofilms in wastewater treatment plants [39]. The observations indicate that the accumulation of nitrite within the low nitrate zone occurs in diverse aquatic environments that harbor redox gradients.

### Accumulated nitrite is likely produced by nitrate reduction but only accounts for a small fraction of the consumed nitrate

The nitrite concentration maxima in the 30 sediment cores (i.e., 2 AMOR sites and 28 reference sites) are generally below 3 µM (Fig. S3), with the maximum nitrite of 8 µM detected in Station 13 of [23] in the Pacific Ocean (Site #15 in Fig. 1D). These nitrite concentrations are comparable or higher than those measured in oxygen deficient zones [e.g., [40, 41]]. In the 30 cores, nitrite concentrations are generally lower than the concomitant nitrate concentrations, indicating that nitrite is only a minor inorganic nitrogen species in the sediments. Yet, nitrite is a central metabolite for many microorganisms, and the low concentrations only imply its fast turnover is well coupled in the environment rather than it is an unimportant metabolite [42].

In the two AMOR cores, the nitrite accumulating zones were well separated from the overlying oxic zones (Fig. 1B, C), indicating that aerobic processes (e.g., ammonia and nitrite oxidation) may not contribute substantially, if at all, to the generation or consumption of the accumulated nitrite. Instead, the accumulated nitrite more likely results from the imbalance between anaerobic processes of nitrite production (e.g., dissimilatory nitrate reduction) and nitrite consumption (e.g., nitrite reduction and anammox).

Nitrite accumulation in the nitrate-depletion zone also indicates that some of the detected nitrite can diffuse both upward and downward and support two distinct zones (e.g., above and below the nitrate depletion depth) that harbor intensified nitrite consumption. By calculating the nitrate influx and the total (the sum of the upward and downward) efflux of nitrite from the nitrate-depletion zone of the total 30 sediment sites shown in Fig. 1D, we found that at all but one site (Site #14, Pacific Station 12 reported in [23]) the nitrate flux is higher than that of the combined nitrite flux at all sites (Fig. 1E). Because of this, the calculated ratio of nitrate to nitrite flux at all but one site is less than 0.6 (Fig. 1F), with an average ratio of 0.285 ± 0.07 (mean ± 95% confidence interval). This calculation suggests that (i) nitrite flux only accounts for on the order of a quarter of the nitrate flux consumed within the nitrate-depletion zone and that (ii) the majority of the nitrate diffusing into that zone is lost by further reduction to unmeasured gaseous compounds (e.g., N_2_).

#### Prevalence of anammox bacteria in GS14-GC04

**Fig. 2:**
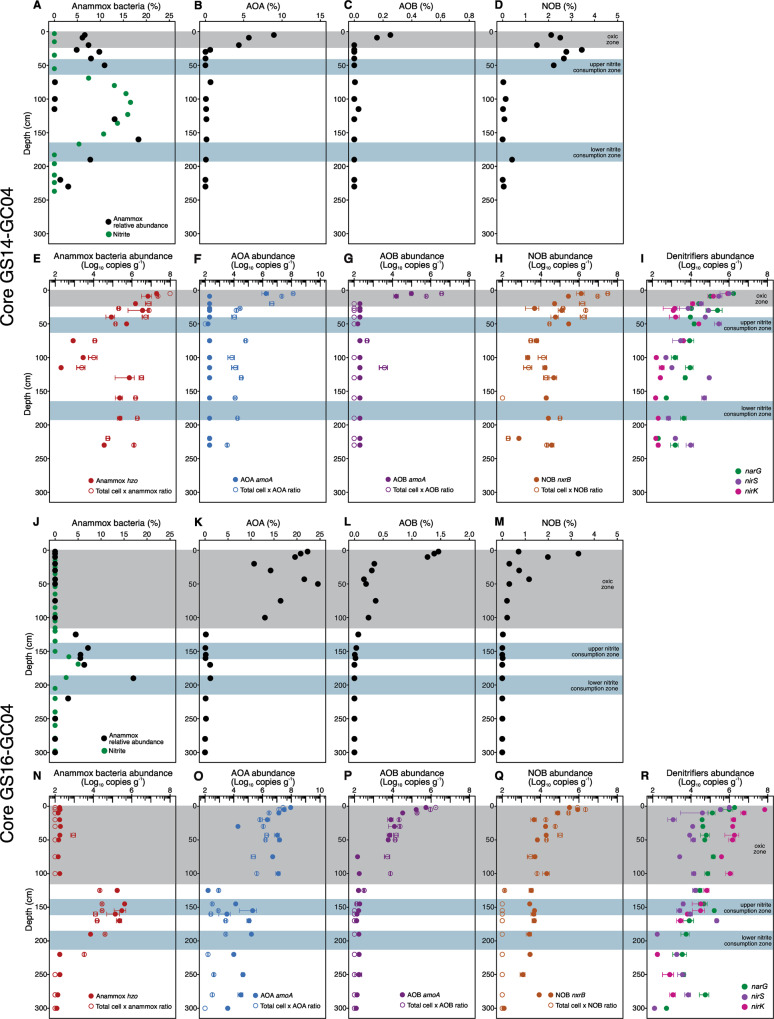
Abundances of anammox bacteria, ammonia-oxidizing archaea (AOA), ammonia-oxidizing bacteria (AOB), nitrite-oxidizing bacteria (NOB), and denitrifying bacteria. Data from both cores GS14-GC04 (**A–I**) and GS16-GC04 (**J–R**) are shown. Data in (**A–D**) and (**J–M**) are relative abundances of the functional groups assessed by 16 S rRNA gene amplicon sequencing. In (**E-I**) and (**N-R**), filled circles indicate the absolute abundances of these groups determined by qPCR using specific primers targeting their diagnostic genes, while open circles denote the absolute abundances of anammox bacteria, AOA, AOB, and NOB calculated as the product of the total cell numbers (shown in Fig. S4A) and their respective relative abundances in the total community. The zones are highlighted according to the definitions presented in Fig. 1B, C, while the nitrite profiles are also re-plotted in (**A**) and (**J**) to help denote the two net nitrite consumption zones in each core. Panels (**J**, **N**, **R**) of core GS16-GC04 derive from data published in ref. [16].

To elucidate which microbial groups play a role in controlling the observed nitrite accumulation, we performed 16 S rRNA gene amplicon sequencing for 13 sediment layers of GS14-GC04, while similar data of GS16-GC04 has been previously generated by [13]. We noted the prevalence of putative anammox bacteria (affiliated to both families *Ca*. Scalinduaceae and *Ca*. Bathyanammoxibiaceae [16]) in most layers of GS14-GC04. Anammox bacteria, notoriously slow growers [43], were sizable contributors of the communities in this core, accounting for 6% of the total community in the uppermost sediments in the oxic zone, and increasing to a first peak of 11% of the total community in the upper nitrite consumption zone (Fig. 2A). After a major collapse in the interval of 75–120 cm, the relative abundance of anammox increased again and reached the second peak of a full ~18% of the total community within the second nitrite consumption zone before again decreasing in deeper sediments (Fig. 2A). By comparison, anammox communities in other systems represent <5% of the total population in hadal sediments [10] and <2% in the Arabian Sea ODZ [44]. The second peak was roughly within the broad nitrate-ammonium transition zone. By contrast, anammox bacteria in GS16-GC04 were mainly detected (up to 18% of the community) within the nitrate-ammonium transition zone (~120–190 cm) but not the oxic zone (Fig. 2J). Still, like GS14-GC04, this second core shows two relative abundance peaks observed in the upper and lower net nitrite consumption zones flanking the nitrite maximum (Fig. 2J).

**Fig. 3:**
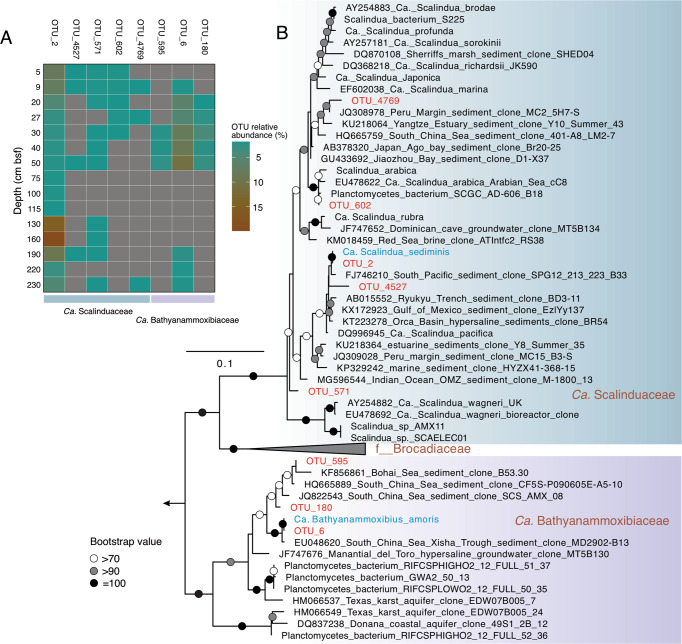
Distribution and phylogeny of anammox bacteria in GS14-GC04. **A** A heatmap showing the occurrence and relative abundance of eight anammox OTUs in the investigated sediment layers. The taxonomic classification of the individual OTUs, showed at the bottom of the heatmap, is based on the phylogenetic placements in (**B**). **B** Maximum-likelihood phylogenetic tree of anammox bacteria. Anammox bacteria OTUs (97% identity cutoff) recovered from GS14-GC04 are highlighted in red. The two genomes recovered from AMOR sediments are highlighted in blue. The bar indicates estimated sequence divergence per residue. The robustness of the tree was evaluated by 1,000 times of ultrafast bootstrap iteration, and bootstrap values higher than 70 are shown by symbols indicated in the legend.

To check whether the relative abundance changes of anammox bacteria are caused by growth/decay of other taxa vs. those of anammox themselves, we tracked the absolute abundances of anammox bacteria in the two AMOR cores using two complementary methods: (i) qPCR of the functional gene *hzo*, which encodes hydrazine dehydrogenase, the ultimate step of the anammox metabolism and therefore a diagnostic gene for anammox bacteria, and (ii) calculation as the product of the total cell abundance (estimated as the sum of the 16 S rRNA genes as presented in Fig. S4A for core GS14-GC04) and the relative abundances given by the 16 S rRNA gene amplicon sequencing. As shown for other AMOR cores [13], results of the two methods generally agree with each other in the two cores (Fig. 2E, N), indicating the major anammox clades are accounted for in this analysis. The prevalence of anammox bacteria in the upper and lower portions of GS14-GC04 was corroborated by their high absolute abundances in the range of 10^6^–10^8^ cells g^−1^ wet sediment, while relatively lower abundances of 10^2^–10^4^ cells g^−1^ are detected in the middle section of the core (75–120 cm bsf) (Fig. 3E). In contrast, anammox bacteria in GS16-GC04 were confined within the nitrate-ammonium transition zone (Fig. 2N), similar to the other three AMOR cores described in [13]. Therefore, our results from GS14-GC04 suggest that anammox bacteria can thrive in marine sediments further from the nitrate-ammonium zone than previously implied.

Anammox bacteria (mainly affiliated with *Ca*. Scalinduaceae) were also detected in the oxic zone (Fig. 2E, with up to 20 µM O_2_) of GS14-GC04. Such presence of anammox bacteria in the presence of oxygen was not detected in GS16-GC04 (Fig. 2N), the other previously reported AMOR cores [13], or hadal trench cores [10]. Although early bioreactor studies have shown that 1 µM O_2_ reversibly inhibits the anammox metabolism [45], anammox bacteria and activity have been detected in oxygenated seawater with up to 25 µM O_2_ [46, 47], which may be facilitated by associating with particles [48] and the microenvironments therein [49] particularly in high organic carbon enivronments. Particles and colonized surfaces are widespread in marine sediments, which can harbor anoxic microniches to greatly expand the anoxic habitats even in bulk oxygenated environments [50, 51]. Therefore, increased anoxic microenvironments in hydrothermal sediments, which typically have larger grain size than typical sediments [52], could enable the presence of anammox bacteria in the bulk oxic surface sediments. Alternatively, the anammox bacteria detected in the oxic zone could be dormant. Nevertheless, the detection of anammox bacteria in the surface sediments do confirm the previous hypothesis that anammox bacteria thriving in subsurface nitrate-ammonium transition zones were seeded from surface sediments [13].

#### Role of anammox bacteria in ammonium and nitrite consumptions

Ammonium is the major fixed nitrogen species present in most of the anoxic sediment porewaters of continental shelves and slopes. In these sediments, ammonium is mainly produced from organic nitrogen degradation and dissimilatory nitrate reduction to ammonium (DNRA) and can be consumed by biological metabolic activities such as aerobic ammonia oxidation and anammox and also biological re-assimilation, albeit the latter should be minimal due to the extremely slow microbial turnover rates. Previous studies have shown that ammonia-oxidizing archaea (AOA) prevail in the oxic zone [29, 53] and anammox bacteria in the nitrate-ammonium transition zone [13], respectively, which may be the major ammonium consumers in their major niches. In GS14-GC04, despite continuous ammonium release from organic matter degradation as evident by the increasing DIC concentrations with depth (Fig. S2), ammonium was not detected until both nitrate and nitrite were depleted from the porewater (Fig. 1B), suggesting active ammonium consumption throughout the upper 180 cm sediments. However, which organisms dominate ammonium consumption in the sediment interval between the depths of oxygen and nitrate depletion, i.e., between the primary niches of AOA and anammox bacteria, is still unclear.

To better understand the relative importance of anammox bacteria for ammonium consumption, we examined, in addition to anammox bacteria themselves, the distribution (i.e., both the relative and absolute abundances) of AOA and ammonia-oxidizing bacteria (AOB) in the two AMOR cores using the two microbial quantitative methods described above. Consistent with their requirement of oxygen [54, 55], both AOA (affiliated with the class *Nitrosopumilales* [56, 57]) and AOB were mainly detected in the oxic zones (i.e., the upper 10 cm sediments of GS14-GC04 (Fig. 2B, C) and the upper 110 cm of GS16-GC04 (Fig. 2K, L)) by 16 S rRNA gene amplicon sequencing. While AOB of low relative abundances [<0.3% of the total communities throughout GS14-GC04 (Fig. 2C) and <1.5% throughout GS16-GC04 (Fig. 2L)] seem to be restricted to the oxic zones (Fig. 2G, P), AOA were detected not only in the oxic zones but also in deeper anoxic sediments (Fig. 2F, O). The discrepancy of AOA abundances determined by the two methods (Fig. 2F) may be attributed to the possibility that the qPCR primers of the AOA *amoA* gene assays fail to detect some novel AOA genotypes and therefore underestimate the AOA abundances. Although AOA have the potential to oxidize ammonium to nitrite in the absence of oxygen [58], their abundances in the sediment interval between the depths of oxygen-depletion and nitrate-depletion were at least one order of magnitude lower than those of anammox bacteria, making it plausible that anammox bacteria dominate the ammonium consumers across anoxic depths. Therefore, in addition to the nitrate-ammonium transition zone [13], ammonium liberated from organic matter degradation in sediments between the depths of oxygen-depletion and nitrate-depletion of GS14-GC04 may also be consumed predominantly by anammox bacteria as a dissimilatory substrate and by all microbes as their assimilatory nitrogen source.

To support our speculation that dissimilatory nitrate reduction was likely the process of nitrite generation in the anoxic sediments of both AMOR cores, we detected and quantified the abundance of nitrate reducing bacteria by qPCR targeting the *narG* gene encoding the membrane-bound nitrate reductase alpha subunit. We detected *narG* throughout the cores, which generally showed a downcore decreasing trend. In particular, we detected up to 10^6^ copies g^−1^ of *narG* in the uppermost sediments and ~10^4^ copies g^−1^ of *narG* within the nitrite accumulating zones of the two AMOR cores (Fig. 2I, R), suggesting that nitrate reducers may employ this pathway to reduce nitrate and therefore produce the accumulated nitrite.

To assess the contribution of anammox bacteria to nitrite consumption, we also quantified the contemporaneous distributions of nitrite-oxidizing and nitrite-reducing bacteria, the other two functional groups involved in nitrite consumption. In both GS14-GC04 and GS16-GC04, the relative abundance of NOB affiliated with the bacterial genera *Nitrospira* and *Nitrospina* were observed to increase with depth in the shallow sediments, and then decrease to low levels (<0.5% of the total communities) in sediments without detectable oxygen (Fig. 2D, M). The presence of putative NOB in anoxic sediments is also supported by the calculated absolute abundances (Fig. 2H, Q). These observations suggest that some NOB may persist in anoxic sediments for long periods of time. Although *Nitrospira* and *Nitrospina* NOBs are metabolically versatile [e.g., as reviewed in [59]], they are not known to maintain nitrite oxidation activity without oxygen and therefore should not greatly affect the distribution of nitrite in anoxic sediments. Moreover, the abundances of nitrite-reducing bacteria, as indicated by the absolute abundances of *nirS* and *nirK* genes, were at least one order of magnitude lower than those of anammox bacteria (Fig. 2I, R). In the nitrite accumulating zones of both cores, the nitrite reducing bacterial populations were dominated by *nirS*-containing members (Fig. 2I, R). Compared to the adjacent layers, the nitrite accumulating zones in both cores harbored higher rather than lower abundances of *nirS* (Fig. 2I, R), indicating that the accumulated nitrite does not result from an abundance decrease of nitrite reducers. However, because gene abundance variations do not necessarily represent metabolic rate differences, future rate measurements of nitrite reduction across various depths are required to reliably assess the impact of nitrite reducers on the distribution of nitrite in the AMOR sediments. Nevertheless, from abundance arguments alone anammox bacteria are crucially important, outnumbering other dissimilatory ammonium and nitrite consumers by at least an order of magnitude at the depths of nitrite accumulation.

#### Identities and distribution of anammox bacteria in GS14-GC04

To elucidate the reasons leading to the two relative abundance peaks of anammox bacteria in GS14-GC04, we examined the anammox bacteria community at the level of individual OTUs (97% nucleotide identity cutoff). Anammox bacteria were represented by 8 OTUs (OTU_2, OTU_6, OTU_180, OTU_571, OTU_595, OTU_602, OTU_4527, and OTU_4769) (Fig. 3A). Among these anammox phylotypes, only OTU_2 was detected throughout the sediment core, while the other OTUs were only detected in discrete sediment horizons (Fig. 3A). Phylogenetic analysis (Fig. 3B) indicated that OTU_2, OTU_571, OTU_602, OTU_4527, and OTU_4769 are members of the *Ca*. Scalinduaceae family, with OTU_2 matching with *Ca*. Scalindua sediminis, an anammox bacterium previously proven to be prevalent in AMOR sediments [13]. OTU_602 and OTU_4769 fell into the broad cluster containing *Ca*. S. brodae [60], *Ca*. S. profunda [15], and *Ca*. S. japonica [14], three anammox enrichment cultures from coastal sediments. The other three OTUs (OTU_6, OTU_180, and OTU_595) are members of the newly proposed anammox bacterial family *Ca*. Bathyanammoxibiaceae [16], and cluster with uncultured anammox bacteria from the AMOR area [13] and other locations such as the South China Sea [61] (Fig. 3B). Analyses of the identities and distribution of anammox bacteria in GS16-GC04 were previously described elsewhere [16], in which members of both families of *Ca*. Scalinduaceae and *Ca*. Bathyanammoxibiaceae were also found.

#### Long-term niche partitioning between the two anammox families and its co-occurrence with nitrite accumulation

**Fig. 4:**
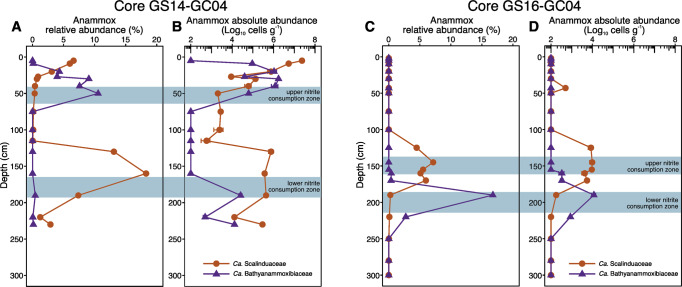
Niche partitioning of anammox bacterial families in GS14-GC04 and GS16-GC04. Relative abundances of the anammox families (*Ca*. Scalinduaceae and *Ca*. Bathyanammoxibiaceae) throughout cores GS14-GC04 (**A**) and GS16-GC04 (**C**), as assessed by 16 S rRNA gene amplicon sequencing. Absolute abundances of the two anammox families in cores GS14-GC04 (**B**) and GS16-GC04 (**D**), calculated as the product of the total cell numbers times their relative abundances in the total communities. Panels (**C**, **D**) are replotted from ref. [16].

Anammox bacteria of the two families exhibit markedly contrasting distribution patterns in both AMOR cores. In GS14-GC04, *Ca*. Scalinduaceae accounted for 7% of the total community in the shallowest sediment and decreased with depth until increasing again in the interval of 120–220 cm, with the peak (18% of the total community) detected at 160 cm (Fig. 4A). *Ca*. Bathyanammoxibiaceae showed the opposite trend. This family was undetectable in the two uppermost examined sediment layers, but increased in the upper sediments to reach the peak (11% of the total community) at 50 cm, before decreasing to low levels in deeper layers (Fig. 4A). In GS16-GC04, *Ca*. Scalinduaceae occupied the interval of 125–170 cm (i.e., the upper portion of the nitrate-ammonium transition zone), while *Ca*. Bathyanammoxibiaceae was confined in the interval of 170–220 cm (i.e., the lower portion of the nitrate-ammonium transition zone) (Fig. 4C). Such distribution of anammox bacterial families observed in GS16-GC04 was also visible in GS16-GC05 (Fig. S5), another AMOR core described previously in [13], in which weak signals of nitrite’s presence in the interval of 50–60 cm were noted but not quantified during the onboard measurements. These observations in the AMOR cores provide the first evidence of niche partitioning (trading between dominant families) between the two anammox bacterial families in the marine environment.

Differentiating the two anammox bacterial families is helpful to better evaluate their respective roles in nitrite consumption. By calculating the absolute abundances of *Ca*. Scalinduaceae and *Ca*. Bathyanammoxibiaceae, it is clear that their absolute abundances peaks match well with the two net nitrite consumption zones above and below the nitrite concentration maxima in GS14-GC04 (Fig. 4B) and GS16-GC04 (Fig. 4D), indicating that they are likely contributing substantially to the local nitrite consumption. In the other two AMOR cores (GS14-GC08 and GS14-GC09) where no nitrite accumulation was found [13], no clear niche partitioning between *Ca*. Scalinduaceae and *Ca*. Bathyanammoxibiaceae can be observed [16]. This comparison of a small number of AMOR cores suggests a co-occurrence between nitrite accumulation and the niche partitioning between the two anammox bacterial families in AMOR sediments. While the anoxic depths with low abundance of anammox coincide with nitrite accumulation and are sandwiched between the peaks of the two families, the full dynamics leading to the niche separation of the two anammox bacterial families remain to be clarified with further study.

Regarding the distribution of the two anammox bacterial families, it appears that an opposite trend exists between GS14-GC04 and the other two cores (GS16-GC04 and GS16-GC05): *Ca*. Bathyanammoxibiaceae occupied the upper nitrite consumption zone of GS14-GC04 but the lower ones of GS16-GC04 and GS16-GC05 (Fig. 4B, D, and S5). However, the discrepancy between these cores may be caused by the insufficient coring of GS14-GC04. Although not easily reflected by the relative abundance profile (Fig. 4A), *Ca*. Bathyanammoxibiaceae in GS14-GC04 showed increases in absolute abundance with depth (including the lower nitrite consumption zone) toward deeper sediments (Fig. 4B). It is possible that its dominance in deeper sediments was not well resolved, because only the onset of *Ca*. Bathyanammoxibiaceae in the deep ammonium-bearing sediments was captured (Fig. 4B). Therefore, in the AMOR cores examined here, we speculate that *Ca*. Bathyanammoxibiaceae likely prefers conditions of higher ammonium availability and *Ca*. Scalinduaceae lower ammonium conditions.

Low activity of microbes in subsurface sediments results in long generation times and can prolong the population evolutionary process. The observed abundance maxima of the two anammox bacterial families in GS14-GC04 and GS16-GC04 were separated by ~110 cm and 45 cm of sedimentation, respectively (Fig. 4). Given the sedimentation rate of ~2 cm ky^−1^ at this area [62], the maximum duration of the niche partitioning between the two anammox families in the two AMOR cores can be estimated to be about 55,000 years. The partial collapse of the whole anammox bacterial population in GS14-GC04 observed during this prolonged process of niche partitioning (Fig. 2E) can be caused by the changes of the two essential substrates of anammox bacteria: nitrite and ammonium. However, the following two observations speak against the scenario that the slight increase of nitrite in the nitrite accumulation zone can strongly affect the activity or abundance of anammox bacteria. First, considering the observation that anammox bacteria abundance was higher in the low nitrite depths but lower in the high nitrite depths (Fig. 2A, E), it is unlikely the measured nitrite concentrations are too low to fuel the detected anammox bacteria. Second, the highest nitrite concentrations measured in GS14-GC04 (3.3 µM) is much lower than the reported mM levels of tolerable nitrite by anammox bacteria (e.g., 7.5 mM for *Ca*. S. japonica [63], 2.1 mM for *Ca*. Kuenenia stuttgartiensis [64], and 6 mM for anammox bacteria enriched from wastewater sludge [65]), indicating that the local nitrite concentrations should not inhibit the anammox bacteria. Instead, decreased ammonium supply is a plausible factor responsible for the partial collapse of the anammox bacterial population. Comparing to the nitrate-depletion zone, shallower sediments may receive higher ammonium supply due to the higher organic matter degradation rates, while deeper sediments may also have higher ammonium supply due to the upward diffusion of ammonium from deeper anoxic sediments. The lower ammonium supply in the nitrite accumulation zone may have limited the anammox population in GS14-GC04 and therefore sustained the accumulation of nitrite. Compared to GS16-GC04, GS14-GC04 features a higher magnitude of nitrite accumulation (Fig. 1C), greater vertical partitioning between the anammox families (Fig. 4), and a clear anammox population collapse (Fig. 2N), which can be attributed to the extended separation between nitrate and ammonium (Fig. 1B). Different from GS14-GC04, ammonium diffusing from deep sediments of GS16-GC04 is not only consumed within the lower nitrite consumption zone but also can enter the nitrite accumulation zone (Fig. 1C) and support the anammox bacteria residing there. In other words, when the two different ammonium sources are too far apart to support anammox in the middle, nitrite can accumulate, with more profound effects in GS14-GC04 than GS16-GC04. Due to the reliance on the vertical separation between nitrate and ammonium, the further these two nutrients are split, the more nitrite should accumulate, as is observed with GS14-GC04 vs. GS16-GC04.

#### Potential mechanisms driving the observed anammox niche partitioning

**Fig. 5:**
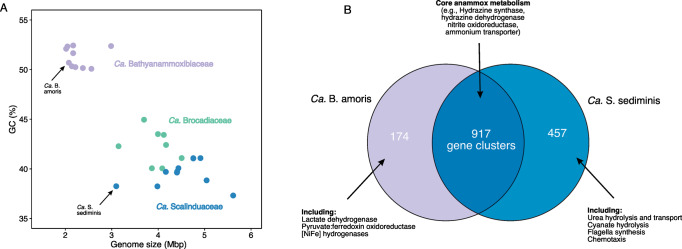
Comparative analysis of genomes of the dominant anammox bacteria in marine sediments. **A** A plot of genome size against GC content of the three families of anammox bacteria genomes. *Ca*. Bathyanammoxibius amoris (in this study) and *Ca*. Scalindua sediminis (Ref. [13]), representatives of the families *Ca*. Bathyanammoxibiaceae and *Ca*. Scalinduaceae widespread in marine sediments, are highlighted. **B** Venn diagram showing the shared and unique gene clusters between *Ca*. B. amoris and *Ca*. S. sediminis.

Given the lack of anammox cultures from pelagic marine sediments, we relied on comparative genomic analysis to identify potential (and probable) reasons that lead to the niche partitioning between the two anammox bacterial families in AMOR sediments. High quality genomes are a prerequisite for such analysis. Although *Ca*. Scalindua sediminis [13] is a high-quality representative of the *Ca*. Scalinduaceae family, the previous metagenome-assembled genome (MAG) of *Ca*. Bathyanammoxibiaceae in AMOR sediments, Bin_158, was estimated to be only 74% complete [16]. Therefore, to obtain high-quality representative genomes of *Ca*. Bathyanammoxibiaceae in AMOR sediments, we performed metagenome sequencing on the sediment horizon GC05_55cm, because *Ca*. Bathyanammoxibiaceae in this sediment layer was revealed to account for 28% of the total prokaryotic community by 16 S rRNA gene amplicon sequencing [16]. By metagenome assembly and binning, we obtained a high-quality MAG (96.6% completeness and 1.5% redundancy) affiliated with *Ca*. Bathyanammoxibiaceae. The contigs of this MAG show higher guanine-cytosine (GC) contents than the co-occurring *Ca*. Scalindua sediminis (Fig. 5A and S6), and therefore can be reliably distinguished. This MAG is 2.1 mega-base pairs in size, smaller than anammox bacterial of other families (Fig. 5A), and with 1905 coding genes distributed on 32 scaffolds. It has an average nucleotide identity of 98% with Bin_158 previously recovered from core GS14-GC08 [16] and therefore can be regarded as the same anammox bacterial species shown to prevail in AMOR sediments. It has a ribosomal operon, and the 16 S rRNA gene (1 334 bp) is a 100% match with OTU_6 of GS14-GC04 presented here (Fig. 3B) and with OTU_23 of the four previously characterized AMOR cores [16], indicating that it can represent the most dominant Bathyanammoxibius phylotype in these AMOR cores. It also contains all necessary genes for the core anammox metabolism, including hydrazine synthase (though the alpha, beta, and gamma subunits are located at the ends of two separated contigs), hydrazine dehydrogenase, and nitrite oxidoreductase. We provisionally name this MAG *Candidatus* Bathyanammoxibius amoris (named after AMOR, the originating location of this MAG).

Using *Ca*. S. sediminis and *Ca*. B. amoris as representative genomes of the *Ca*. Scalinduaceae and *Ca*. Bathyanammoxibiaceae families, respectively, shown to dominate in this system, we performed a comparative genomic analysis to identify potential reasons that may lead to the niche partitioning between the two anammox bacterial families in AMOR sediments. The two genomes combined contain 4808 genes summarized in 1548 gene clusters, of which 917 are shared by the two genomes (Fig. 5B). Of the remaining 631 gene clusters, 457 are unique in *Ca*. S. sediminis, and the other 174 gene clusters are unique in *Ca*. B. amoris (Fig. 5B). Since both are anammox genomes, genes encoding the key enzymes of the core anammox metabolism are among the shared gene clusters (included in Supplementary dataset S2). Comparing to *Ca*. Scalindua sediminis [13], *Ca*. B. amoris lacks urease and cyanase (Fig. 5B), indicating that it does not have the capacity to conserve energy or produce extra ammonium from the degradation of urea and cyanate. Although cyanate availability in marine sediments has not been determined, urea concentrations had been measured to be eight times lower than ammonium [66, 67]. The majority of the urea production in anoxic sediments attributed to microbial degradation [68] of purines and pyrimidines [69], while in oxic sediments macrofauna, if exist, may also play a role in urea production [70]. The urea hydrolysis capacity may provide *Ca*. S. sediminis a competitive advantage to live in environments of limited ammonium, such as the surface oxic sediments (Fig. 4A, B). *Ca*. B. amoris also lacks thiosulfate reductase, an enzyme present in *Ca*. S. sediminis and also some other anammox bacteria [71] which may enable them to utilize thiosulfate as an electron acceptor. Unique genes present in *Ca*. B. amoris include genes encoding for lactate dehydrogenase, pyruvate:ferrodoxin oxidoreductase and [NiFe] hydrogenase (Fig. 5B), all of which may be involved in fermentation.

**Fig. 6:**
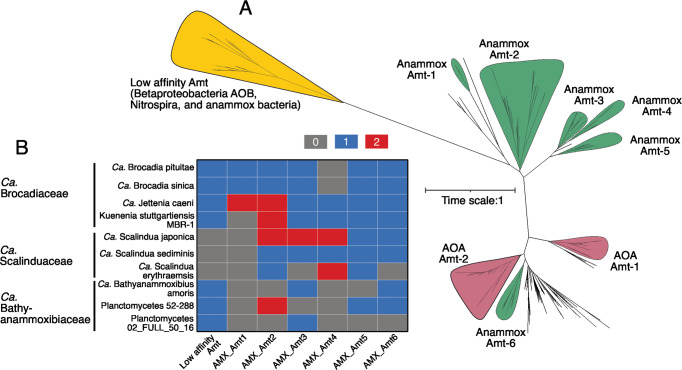
Phylogeny and distribution of ammonium transporters (Amt) in anammox bacteria. **A** Maximum-likelihood phylogenetic tree of Amt in anammox bacteria and other related nitrogen cycling groups (AOB, NOB, and AOA). Amt clades of nitrogen cycling groups are highlighted with different colors. The bar indicates estimated sequence divergence per residue. **B** Heatmap showing the occurrence of Amt in 10 selected high-quality anammox bacterial genomes.

Given that observed ammonium concentrations are profoundly different between the two niches of anammox bacteria, we investigated the types and numbers of ammonium transporters (Amt)—the essential cell apparatus for ammonium assimilation conserved in all domains of life—in available high-quality anammox bacteria genomes. We identified a total of 55 Amt among the 10 selected high-quality anammox genomes. Phylogenetic analysis of Amt suggested that anammox bacteria contain Amt of both Rh-type and MEP-type (Fig. 6A). We identified one clade of anammox Amt in the Rh-type branch clustering with those of AOB and *Nitrospira* NOB [72], and 6 anammox Amt clades in the MEP-type branch (Fig. 6A). Rh-type transporter proteins in AOB [73, 74] and other organisms [75] were demonstrated to have low ammonium affinity and can only be operational in high ammonium concentrations in the millimolar range, while MEP-type ammonium transporters have higher affinity [76, 77] and can be efficient under conditions of low ammonium concentrations. A Rh-type Amt of low affinity is conserved in genomes of the families *Ca*. Brocadiaceae and *Ca*. Bathyanammoxibiaceae, but seem to be absent in *Ca*. Scalinduaceae (Fig. 6B). For the MEP-type, high-affinity Amt, anammox bacteria in the *Ca*. Scalinduaceae family have between four and eight, while *Ca*. Bathyanammoxibiaceae members have only 2–5 of these ammonium transporters (Fig. 6B). Combined with the lack of access to alternative substrates and extra ammonium, encoding fewer high-affinity ammonium transporters in *Ca*. Bathyanammoxibiaceae than *Ca*. Scalinduaceae may drive the former inhabit only conditions of high concentrations or fluxes of ammonium, which is supported by the observed preference of *Ca*. Bathyanammoxibiaceae in sediment layers of (observed or inferred) higher ammonium availabilities.

The genome-inferred preference of higher ammonium availabilities for *Ca*. Bathyanammoxibiaceae is also consistent with the recent phylogenomic and molecular clock analysis of anammox bacteria [78]. In this work, anammox bacteria on Earth were inferred to emerge around the Great Oxidation Event [78], before which ammonium was the dominant oceanic nitrogen species [1]. *Ca*. Bathyanammoxibiaceae is more deep-branching than *Ca*. Scalinduaceae, which could have been more adapted to original conditions (e.g., high ammonium concentrations) of anammox bacteria.

#### Limitations

It is worth noting that microbiological data of only two of the 30 sediment sites that feature nitrite accumulation were analyzed in this study, and whether the proposed mechanism here for the AMOR sediments is applicable to other global sites more broadly remains unclear. Depth-resolved microbiological data are the key to make this assessment. Although microbial communities in some of the 28 literature sites have been characterized, especially those from the Atacama Trench [10, 79], at least two differences can be seen between these Atacama Trench cores and the two AMOR cores investigated here. (i) The relative abundance maxima of anammox bacteria in hadal trench sediments (maximally 5% of the total communities; [10]) are much lower than those in AMOR cores (maximally 15% of the total communities; Fig. 2). (ii) The shapes of nitrite profiles are different. While the upper nitrite consumption zones in both AMOR cores are well separated from the oxic zone (Fig. 1B, C), nitrite was frequently detected in the upper part including the oxic zone of some of the Atacama Trench cores (e.g., AT1, AT3, AT4, AT6, and AT7; Fig. S3), indicating that aerobic processes may play a role in generating or depleting nitrite in shallow sediments of these trench cores. Such differences are to be expected at these disparate sites, each characterized by different depth, organic matter and nutrient supply, and sedimentation rate. Microbiological investigations of more sediment cores are needed to develop a more complete understanding about microbial processes underlying the observed nitrite accumulation in marine sediments.

## Conclusion

We combined biogeochemical, microbiological, and genomic data to study anammox bacteria and their geochemical impacts in marine sediments. We revealed that the anammox community consisted of members of both families *Ca*. Scalinduaceae and *Ca*. Bathyanammoxibiaceae and documented a niche partitioning between them in two sediment cores retrieved from Arctic Mid-Ocean Ridge. These cores showed nitrite accumulation around the nitrate-depletion zones, an analogous feature also observed in 28 other globally distributed marine sediment cores and in other stratified aquatic environments. The accumulated nitrite is mainly produced by nitrate reducers, and accumulates due to limitation of ammonium for anammox bacteria and nitrite reducers. The observed nitrite accumulation in the AMOR sediment cores is accompanied by the niche partitioning between the two anammox bacterial families, in which *Ca*. Bathyanammoxibiaceae and *Ca*. Scalinduaceae occupy higher and lower ammonium conditions, respectively. This niche partitioning is likely driven by the differential capacities in ammonium assimilation and utilizing alternative organic nitrogen substrates like urea and cyanate. Future efforts in developing mechanistic models that can explain the observed geochemical and microbiology data while also reconciling the sedimentation history will greatly advance our understanding of the interactions between the nitrogen cycling processes in marine sediments.

### Etymology description

*Candidatus* Bathyanammoxibius amoris. Bathyanammoxibius amoris (a.mo’ris, N.L. gen. masc, n. *amoris* of AMOR, derived from the oceanographic location (Arctic Mid-Ocean Ridge, AMOR) where this bacterium was found to be abundant). The genome shows 98.8% amino acid identity with Bathyanammoxibius Bin_158 previously reported [16], but is more complete (96.6% compared to 72.4%). It contains essential genes for key enzymes of the anammox metabolism, such as hydrazine synthase, hydrazine dehydrogenase, nitrite oxidoreductase, hydroxylamine oxidoreductase. No urease or cyanase genes were discovered in the genome. The genome reference sequence of *Candidatus* Bathyanammoxibius amoris is JAMXCW000000000. This genome was recovered from core GS16-GC05 (55 cm below the seafloor) of the central Knipovich Ridge (76°55’ N, 7°7.5’ E). The G + C content in the genome is 52.36%.

## Materials and methods

### Study area, sampling, and geochemical measurements

Two cores were studied in this study with the same sampling and analytic procedure, although they were collected during two different cruises. GS14-GC04 (71^o^17.08’N, 6^o^33.69’W), was retrieved using a gravity corer from the seafloor at a water depth of 1050 meters during the CGB 2014 summer cruise onboard the Norwegian R/V G.O. Sars. This coring site is about 50 km west of the Jan Mayen hydrothermal vent field [71.2°N, 5.5°W, [19, 20]] and north of the Jan Mayen fraction zone (Fig. 1A). GS16-GC04 was retrieved using the same method from the east flank of the central Mohns Ridge (72^o^16’ N, 1^o^42’ E). As described elsewhere [13], the retrieved cores were split into two halves on deck. One half was immediately wrapped with plastic films for archiving at 4 ^o^C at the core repository at the University of Bergen, and the other half was used for sampling on the deck. First, the oxygen concentrations were measured using an optode by lowering the sensor into the middle part of selected depths in the working half. The optode sensors were connected to a MICROX TX3 single-channel fiber-optic oxygen meter, which was calibrated according to the manufacturer’s protocols (PreSens, Regensberg, Germany). Second, porewater was extracted using Rhizon samplers [80] from discrete depths. Microbiology subsamples were taken simultaneously with porewater extraction, using sterile 10 ml cut-off syringes from nearly identical depths as the porewater extraction, and immediately frozen at –80 ^o^C for onshore-based DNA analysis.

### Geochemical analyses

Geochemical analyses were performed using the same procedure as described in [13]. Nutrient concentrations in porewater were measured onboard. Concentrations of ammonium (NH_4_^+^), nitrate (NO_3_^–^), nitrite (NO_2_^–^), and dissolved inorganic carbon (DIC) were analyzed colorimetrically by a QuAAtro continuous flow analyzer (SEAL Analytical Ltd, Southampton, UK), following the manufacturer’s protocol. The photometric indophenol method was used for the ammonium measurement [81]. Nitrite was measured as a pink complex after reacting with *N*-1-naphthylethylenediamine dihydrochloride and sulfanilamide. The sum of porewater nitrate and nitrite was measured using the same method after reducing nitrate to to nitrite by a Cu-Cd reduction coil [82]. Nitrate concentrations were calculated as the difference between these two measurements. The protocol for DIC was based on [83]. Porewater samples for metal concentrations (including dissolved Mn and Fe) were acidified by ultrapure nitric acid to a final concentration of 3 vol% and stored in acid-cleaned bottles at 4 ^o^C until analysis. Metal concentrations were determined by Thermo Scientific iCap 7600 ICP-AES (inductively coupled plasma atomic emission spectrometry) at the University of Bergen. For total organic carbon (TOC) and nitrogen (TON) measurements, sediments were first dried at 95 ^o^C for 24 hours and then measured on an element analyzer (Analytikjena multi EA4000, Jena, Germany), after inorganic carbon removal by adding 1 mL of phosphoric acid.

### Diffusive flux calculation

Diffusive fluxes of nitrate into and nitrite effluxes (both upward and downward) from the nitrate-depletion zone in sediment cores were calculated based on the measured profiles using Fick’s first law of diffusion:

$$J = \varphi \cdot D_s \cdot \frac{\partial \left[ C \right]}{\partial z}$$

where, *J* is the flux; φ is the measured sediment porosity; *D*_*s*_ is the sedimentary diffusion coefficient for a given solute (m^2^ yr^−1^) calculated using the *R* package *marelac* [84]; *z* is the sediment depth below the seafloor (m); and *∂[C]/∂z* equals the solute (NO_3_^–^ or NO_2_^–^) concentration gradient (mmol m^−4^), calculated from nearby three data points. The ratio of nitrite to nitrate flux was calculated by dividing the sum of the upward and downward fluxes of nitrite by the (downward) flux of nitrate. The mean value and the 95% confidence interval of this ratio at the 30 sediment sites were calculated in *R*.

### DNA extraction, PCR amplification, and sequencing

Total DNA for amplicon sequencing and qPCR was extracted from ~0.5 g of sediment per sample using the PowerLyze DNA extraction kits (MO BIO Laboratories, Inc.) with the following minor modifications: (1) Lysis tubes were replaced by G2 tubes (Amplikon, Odense, Denmark), and (2) water bathed for 30 min at 60 ^o^C before bead beating (speed 6.0 for 45 s) using a FastPrep-24 instrument (MP Biomedicals). A blank extraction (without sediment addition) was carried out in parallel with the sample extraction batch following the same procedure. The DNA was eluted into 80 µL of molecular grade double-distilled H_2_O (ddH_2_O) and stored at –20 °C until analysis. Amplicon libraries of 16 S rRNA genes were prepared using the primer pair 519 F/806 R in a two-round amplicon strategy [13], with an optimal PCR cycle number in the first round for each sample to minimize over-amplification. Amplicon libraries were sequenced on an Ion Torrent Personal Genome Machine.

### Amplicon sequencing data analysis

Sequencing reads were quality filtered and trimmed to 220 bp using the USEARCH pipeline [85] and chimera were detected and removed using UCHIME. Trimmed reads were clustered into operational taxonomy units (OTUs) at >97% nucleotide sequence identity using UPARSE [86]. Most of the OTUs detected in the extraction blanks (negative controls) were manually removed, except for a few OTUs that may be introduced into the blanks by cross-contamination. Overall, >99.9% of reads in the negative controls were removed. Samples were subsampled to 20,000 reads for each sediment horizon. The taxonomic classification of OTUs was performed using the lowest common ancestor algorithm implemented in the CREST package [87] with the SILVA 138.1 Release as the reference. The relative abundance of anammox bacteria was taken as the total percentage of the OTUs affiliated with the families *Ca*. Scalinduaceae and *Ca*. Bathyanammoxibiaceae [16]. The distribution of individual anammox OTUs was visualized in heatmaps generated using the *R* package *ggplot2* [88].

### Quantification of 16 S rRNA genes and functional genes

Abundances of anammox bacteria were quantified using qPCR by targeting the *hzo* gene (encoding the hydrazine dehydrogenase responsible for the degradation of hydrazine to N_2_) using the primer pair hzoF1/hzoR1 [89] following the procedure described elsewhere [29]. The abundances of denitrifying bacteria were quantified by targeting the *narG* (coding the periplasmic nitrate reductase alpha subunit), *nirS* and *nirK* genes (coding cytochrome *cd1*- and Cu-containing nitrite reductases, respectively), using the protocol described in [29]. The qPCR standards of these functional genes were prepared from PCR amplification of DNA extracts of environmental samples using the corresponding qPCR primers. For *hzo* and *narG* genes, the DNA extracts of a marine sediment horizon (160 cm of core GS14-GC08 [13]) were used, while for *nirS* and *nirK* genes, an Arctic permafrost soil sample was used. After purification, the PCR products were cloned using the StrataClone PCR Cloning Kit (Agilent Technologies, USA), including ligation into vectors and transformation into competent cells of *Escherichia coli* DH5α. The transformed *E. coli* cells were plated onto LB solid medium and grown overnight for the blue/white colony selection. For each gene, a white colony was selected and amplified using the vector primers M13F/M13R, to generate linear qPCR standards. In addition, archaeal and bacterial 16 S rRNA genes were quantified as described in [90]. The qPCR standards for archaeal and bacterial 16 S rRNA gene quantification were genomic DNA of Thaumarchaeota fosmid 54d9 (AJ627422) and *E. coli*, respectively. Total cell abundance was estimated from 16 S rRNA gene copies, assuming a single copy of 16 S rRNA genes in each bacterial or archaeal genome [53]. All gene abundances were determined triplicate in qPCR, and standard deviations are presented using horizontal error bars. Absolute abundances of the aforementioned groups were also calculated as the product of the total cell abundance and the percentage of these groups in the total community assessed by amplicon sequencing.

### Metagenome sequencing, assembly, and binning

To recover high-quality genomes (>90% completeness and <5% redundancy) of *Ca*. Bathyanammoxibiaceae, we focused on the sediment horizon of 55 cm of core GS16-GC05, because our previous survey indicated that this particular sediment horizon harbors the highest relative abundance of *Ca*. Bathyanammoxibiaceae in the total archaea and bacteria community [16]. We extract the total DNA from 6.4 g of sediment (~0.4 - 0.6 g sediment in each of the 12 individual lysis) using PowerLyze DNA extraction kits (MO BIO Laboratories, Inc.) following the manufacturer’s instructions. The DNA extracts were iteratively eluted from the 12 spin columns into 100 µL of ddH_2_O for further analysis.

Shotgun metagenome libraries were constructed using a NEBNext Ultra II FS DNA Library Prep Kit (New England Laboratories) and sequenced (2×150 bp paired-end) on an NextSeq 500 sequencer (Illumina) at the MIT BioMicro Center. The quality of the reads and the presence of adaptor sequences were first checked using FastQC v.0.11.9 [91]. Adapters were removed and reads trimmed using BBDuk implemented in the BBMap package [92]. The overall quality of processed reads was evaluated in a final check with FastQC v.0.11.9 [91], to ensure only high-quality reads (i.e., with a minimum length of 50 bp and a Phred quality score higher than 30) were used in the downstream analysis. The quality-controlled reads were *de novo* assembled into contigs using Megahit v.1.1.2 [93] with the *k*-mer length varying from 27 to 117 and a contig length threshold of 1000 bp. Contigs were grouped into genome bins using three programs: MaxBin2 v2.2.6 [94], MetaBAT v2.15.3 [95], and CONCOCT v1.1.0 [96], all with the default parameters. The resulting bins from these three programs were subject to dereplication and aggregation by DAS_Tool v1.1.4 [97] with the default parameters. The quality of the obtained genome bins was assessed using the option “lineage_wf” of CheckM v.1.1.3 [98]. To improve the quality of the genomes affiliated to the Brocadiales order, the quality-controlled reads were mapped onto the contigs using BBmap [92], and the mapped reads were re-assembled using SPAdes v.3.14.0 [99]. After removal of contigs shorter than 1000 bp, the resulting scaffolds were visualized and re-binned manually using gbtools [100] as described in [13]. The quality of the resulting *Ca*. Bathyanammoxibius genome was checked using the CheckM “lineage_wf” command again, based on the Planctomycetes marker gene set.

### Comparative genomic analysis

We performed a comparative analysis on the genomes *Ca*. Scalindua sediminis [13] and *Ca*. Bathyanammoxibius amoris (recovered in this study), the dominant species of the two anammox bacterial families in marine sediments [16]. We did the analysis using Anvio v7.1 [101] according to the workflow described at http://merenlab.org/2016/11/08/pangenomics-v2/. All genomes were first annotated using Prokka v.1.14 [102] and BLASTp using the Clusters of Orthologous Groups of proteins (COG) [103] as the reference database. The comparative genomic analysis use BLAST to quantify the similarity between each pair of genes, and the Markov Cluster algorithm (MCL) [104] (with inflation parameter of 2) to resolve clusters of homologous genes. The shared and unique genes in the two genomes were identified via the functional enrichment analysis [105].

### Phylogenetic analyses

A maximum-likelihood phylogenetic tree based on 16 S rRNA genes was reconstructed for known anammox bacteria and close relatives of the putative anammox OTUs identified via BLASTn [106] in the NCBI database. Sequences were aligned using MAFFT-LINSi [107] and the maximum-likelihood phylogenetic tree was inferred using IQ-TREE v.1.5.5 [108] with GTR + F + R5 as the best-fit substitution model selected by ModelFinder [109]. 1000 ultrafast bootstraps iterations were performed using UFBoot2 [110] to assess the robustness of tree topology.

For the phylogeny of Amt (ammonium transporter), the sequences of anammox genomes were extracted from the Prokka annotation and used as the queried in BLASTp [106] searches against the NCBI database (>50% similarity were retained), to identify its close relatives. These sequences were complemented with known nitrifiers (e.g. ammonia-oxidizing bacteria (AOB) from the genera of *Nitrosospira, Nitrosomonas, Nitrososcoccus*, nitrite-oxidizing bacteria (NOB) from *Nitrospira* and *Nitrospina*, and ammonia-oxidizing archaea (AOA) from the *Thaumarchaeota* phylum) and aligned using MAFF-LINSi [107]. The alignment was trimmed using trimAl [111] with the mode of “automated”. A maximum likelihood phylogenetic tree was reconstructed using IQ-TREE v.1.5.5 [108] with the LG + F + R7 as the best-fit substitution model and 1,000 ultrafast bootstraps.

## Supplementary information

Supplementary Information

Dataset S1

Dataset S2

Dataset S3

## Data Availability

All sequencing data used in this study are available in the NCBI Short Reads Archive under the project number PRJNA854201. Raw metagenome sequencing data of core GS16-GC05 (55 cm) is available in the NCBI database under the BioSample number SUB11625283. The genome of *Ca*. Bathyanammoxibius amoris is available under the accession number JAMXCW000000000. Raw geochemical data of core GS14-GC04 can be found in Supplementary data S1. A compilation of the porewater profiles of nitrate, nitrite, and ammonium for the 28 reference sites shown in Figure S3 can be found in Supplementary data S2.
