# Supplementary material for: Nitrite accumulation and anammox bacterial niche partitioning in Arctic Mid-Ocean Ridge sediments
Source: ISME Commun. 2023 Mar 29;3:26. doi: 10.1038/s43705-023-00230-y (PMC10060263; doi:10.1038/s43705-023-00230-y)
Supplement: Supplementary file 1 — Supplementary Information [file 43705_2023_230_MOESM1_ESM.docx]

**Supplementary Information**

**Supplementary Text**

***Note 1: The uppermost surface sediment layer of GS14-GC04 may have been lost during coring***

Compared to the four AMOR with significant hydrothermal influences as reported in (Zhao et al., 2020), the uppermost surface sediment layer of GS14-GC04 features (i) a lower concentration of oxygen (15 µM, rather than 150 - 200 µM as those measured in the four AMOR cores previously reported in (Zhao et al., 2020)), and (ii) a higher concentration of DIC (2.75 mM, rather than 2.2 - 2.3 mM reported in (Zhao et al., 2020)). The combination of these observations suggested that the real surface sediments were very likely lost during coring. The exact length of the lost core is difficult to determine based on reconciling geochemical profiles from multiple cores, because so far only one core has been recovered from this area. However, using the same gravity coring device, previous studies have reported < 17 cm core-top lost during deep-sea sediments coring (Chong et al., 2018; Lund et al., 2018).

**Supplementary Figures**


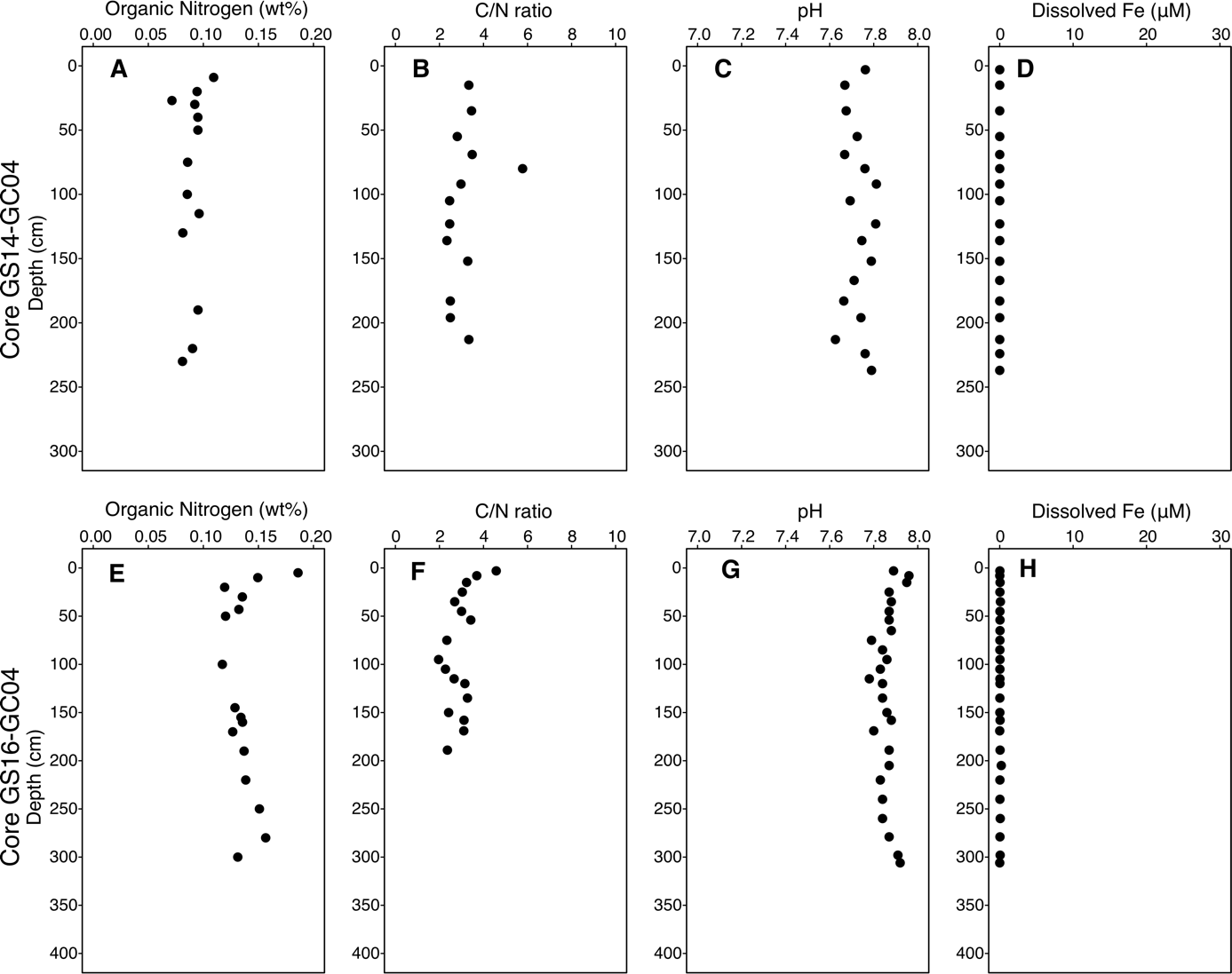


**Fig. S1. Organic nitrogen content (A, E), organic C/N ratio (B, F), porewater pH (C, G), and dissolved Fe concentration (D, H) measured in GS14-GC04 and GS16-GC04.**

**
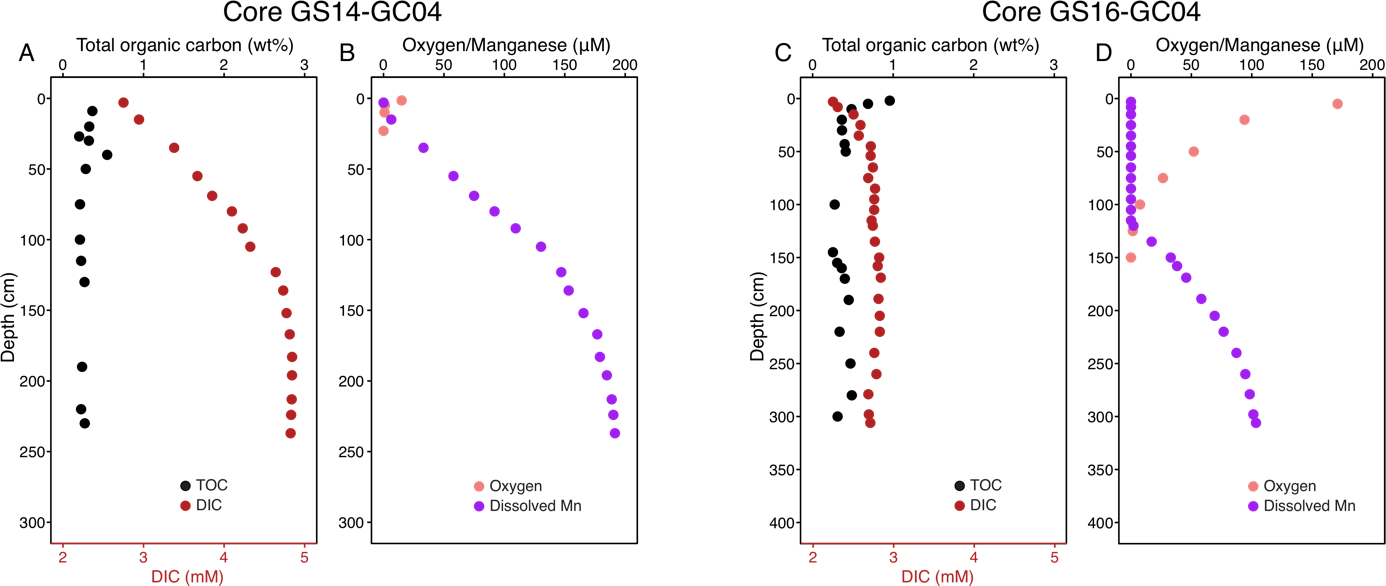
**

**Fig. S2. Geochemical profiles of GS14-GC04 (A, B) and GS16-GC04 (C, D).** Depth profiles of total organic carbon (TOC) and dissolved inorganic carbon (DIC) (**A, C**), oxygen and dissolved Mn (**B, D**).

**
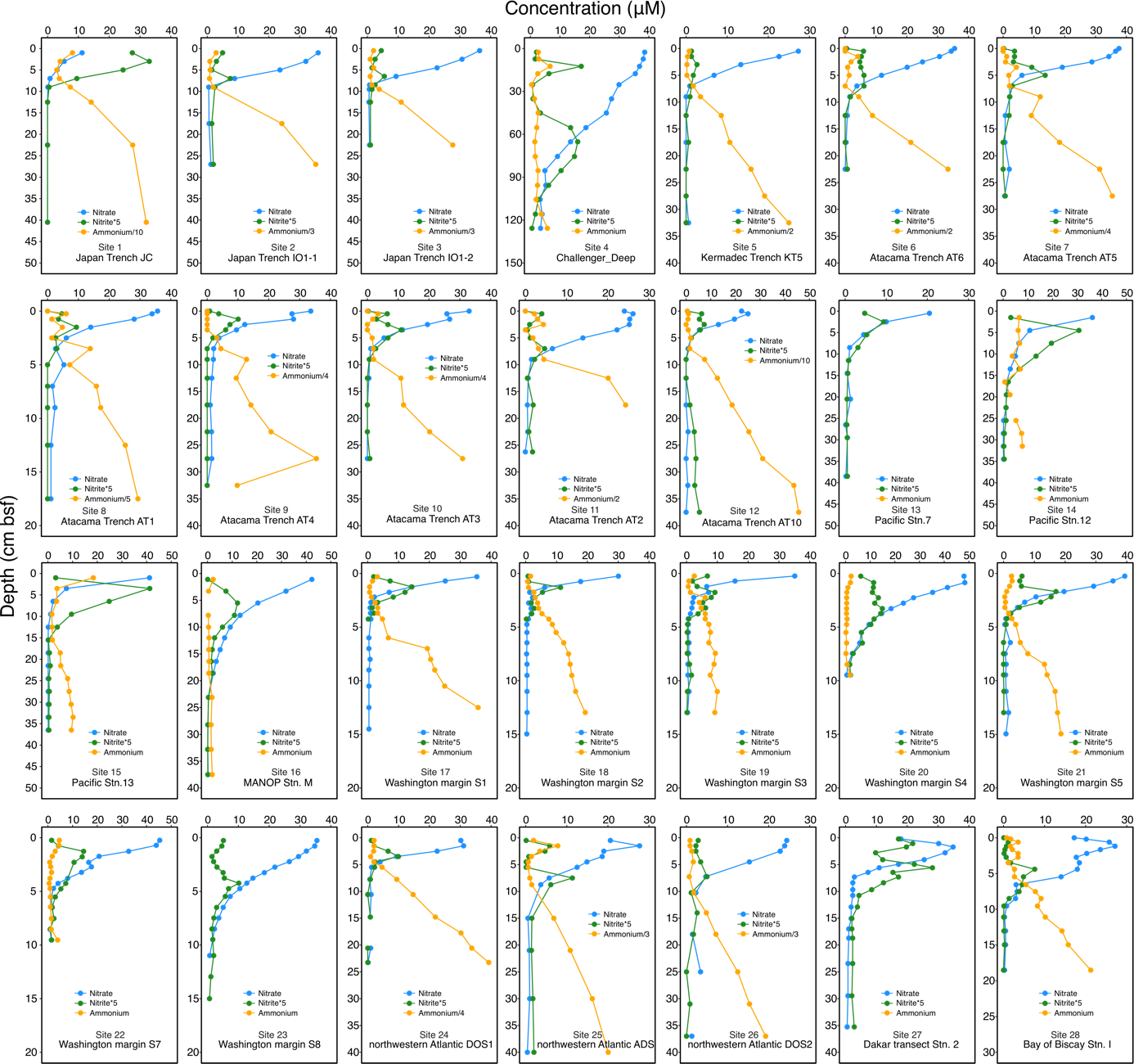
**

**Fig. S3. Porewater profiles of nitrate (blue), nitrite (×5, green), and ammonium (orange) in marine sediment cores where nitrite accumulation in the nitrate-depletion zone is observed.** Ammonium profiles in some cores are not available and therefore are absent in the plots. See Table S2 for the corresponding references and Table S4 for the data.

**
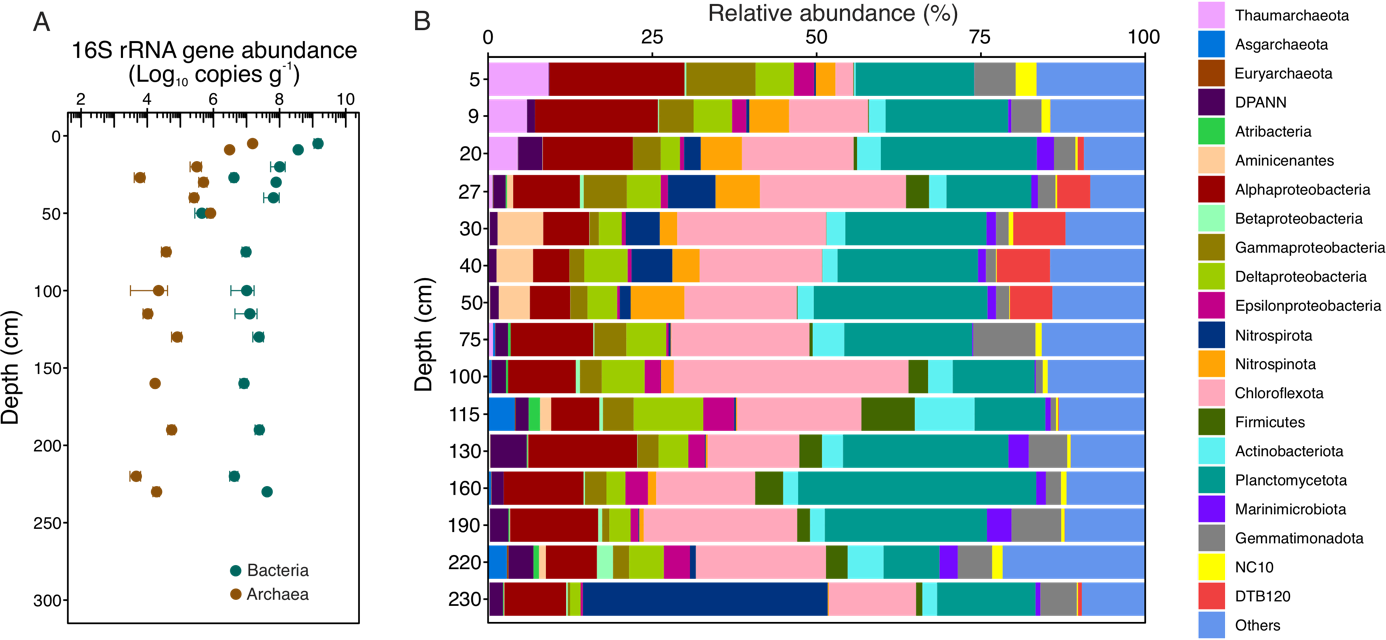
**

**Fig. S4. Abundance (A) and community structure (B) of archaea and bacteria in GC04. (A)** Abundances of archaea and bacteria determined by qPCR using domain-specific primers**.** Dots show the mean values and error bars denote the standard deviations of triplicate measurements. **(B)** Community structure assessed by 16S rRNA gene amplicon sequencing. Minor taxa (with relative abundances of <1%) were grouped into the category “Others”.


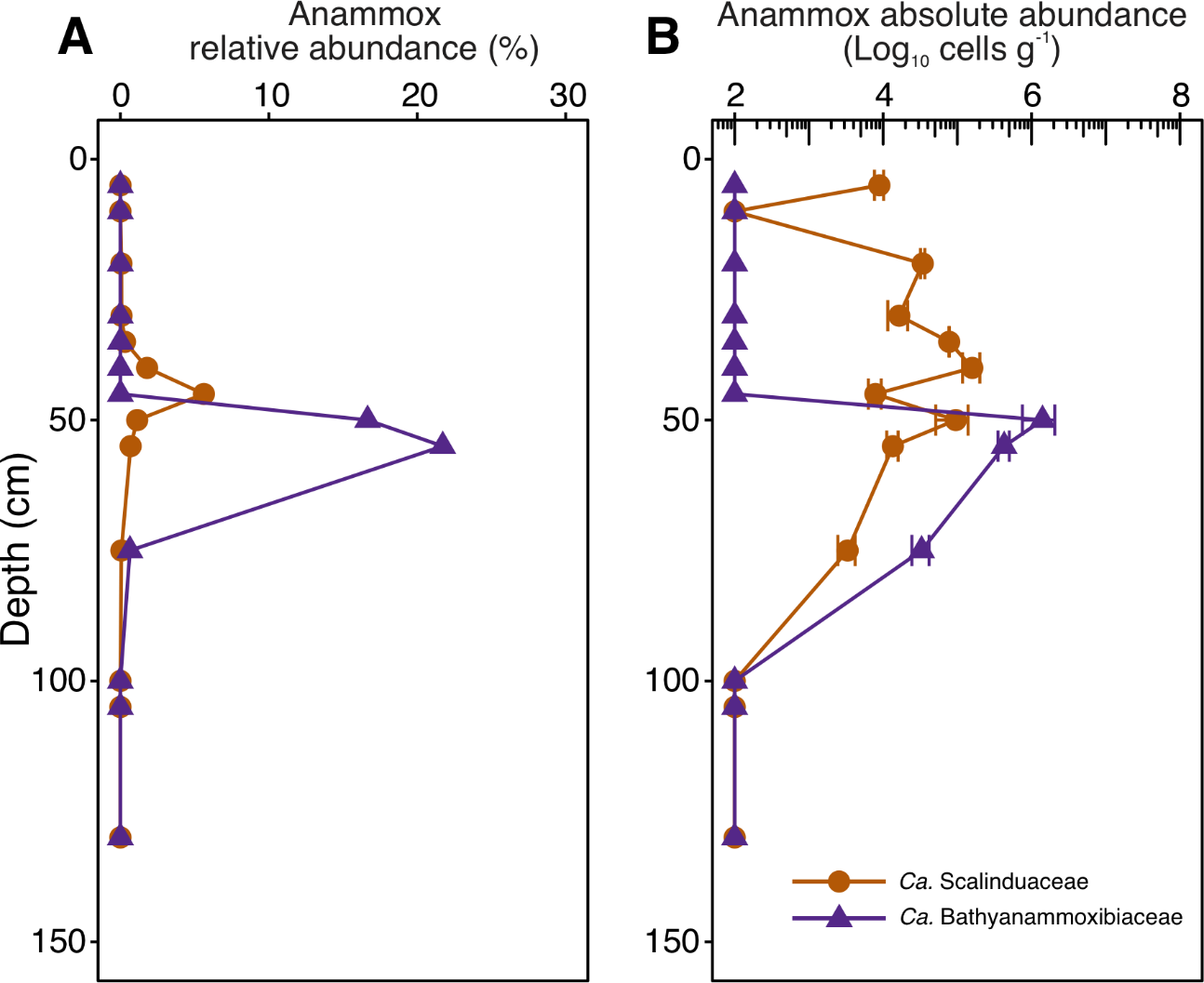


**Fig. S5. Relative (A) and absolute (B) abundances of two anammox bacterial families in core GS16-GC05.** Weak signals of nitrite’s presence in the interval of 50-60 cm was noted but unfortunately not quantified during the onboard measurements. These are replots of data presented in (Zhao et al., 2022), with only the top 1.5 meter of sediments shown, to make the niche partitioning of the two anammox bacterial families more visible.

**
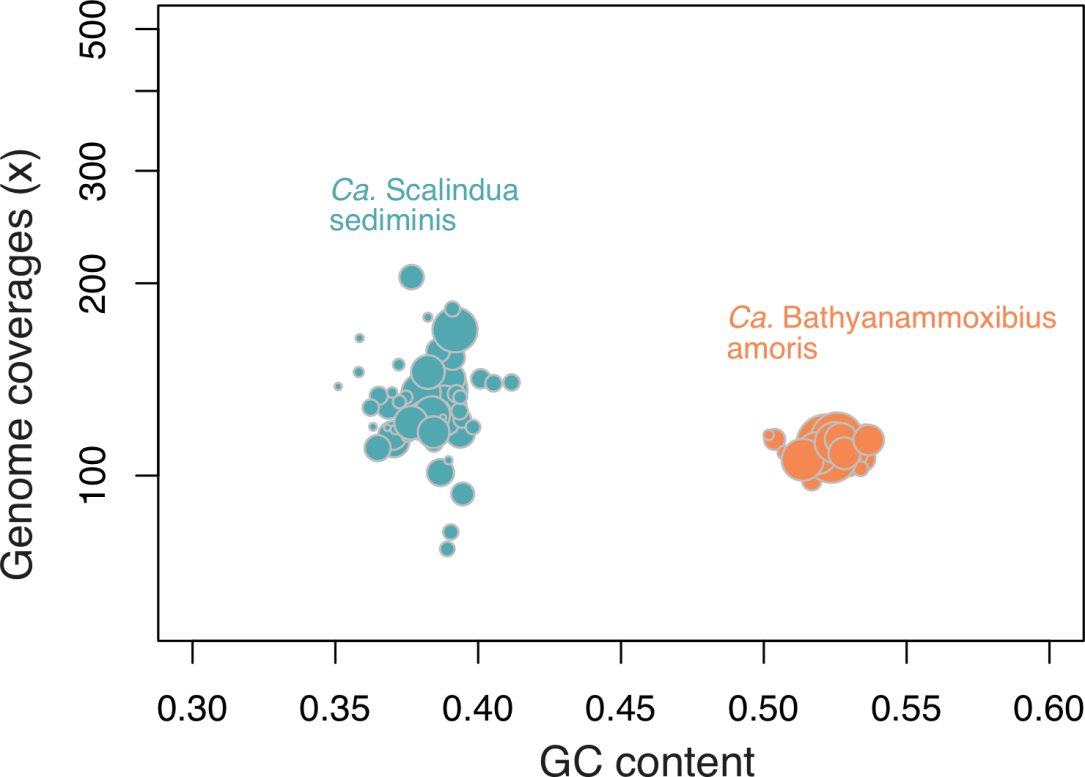
**

**Fig. S6.** **A plot showing the coverages (in the sediment of 55 cm in core GC05) and GC contents of the contigs of *Ca.* Scalindua sediminis and *Ca.* Bathyanammoxibius amoris genomes.**

**Table S1 List of the sediment sites where nitrite accumulation in the nitrate-depletion zone is observed.**

| Site # | Sediment core name | Reference |
| --- | --- | --- |
| 1 | Japan Trench JC | (Hiraoka et al., 2020) |
| 2 | Japan Trench IO1-1 | (Hiraoka et al., 2020) |
| 3 | Japan Trench IO1-2 | (Hiraoka et al., 2020) |
| 4 | Challenger Deep | (Nunoura et al., 2018) |
| 5 | Kemadec Trench KT5 | (Thamdrup et al., 2021) |
| 6 | Atacama Trench AT6 | (Thamdrup et al., 2021) |
| 7 | Atacama Trench AT5 | (Thamdrup et al., 2021) |
| 8 | Atacama Trench AT1 | (Thamdrup et al., 2021) |
| 9 | Atacama Trench AT4 | (Thamdrup et al., 2021) |
| 10 | Atacama Trench AT3 | (Thamdrup et al., 2021) |
| 11 | Atacama Trench AT2 | (Thamdrup et al., 2021) |
| 12 | Atacama Trench AT10 | (Thamdrup et al., 2021) |
| 13 | Pacific Station 7 | (Jahnke et al., 1982) |
| 14 | Pacific Station 12 | (Jahnke et al., 1982) |
| 15 | Pacific Station 13 | (Jahnke et al., 1982) |
| 16 | MANOP Station M | (Emerson et al., 1980) |
| 17 | Washington margin S1 | (Engstrom et al., 2009) |
| 18 | Washington margin S2 | (Engstrom et al., 2009) |
| 19 | Washington margin S3 | (Engstrom et al., 2009) |
| 20 | Washington margin S4 | (Engstrom et al., 2009) |
| 21 | Washington margin S5 | (Engstrom et al., 2009) |
| 22 | Washington margin S7 | (Engstrom et al., 2009) |
| 23 | Washington margin S8 | (Engstrom et al., 2009) |
| 24 | Northwestern Atlantic DOS1 | (Christensen and Rowe, 1984) |
| 25 | Northwestern Atlantic ADS | (Christensen and Rowe, 1984) |
| 26 | Northwestern Atlantic DOS2 | (Christensen and Rowe, 1984) |
| 27 | Dakar transect Station 2 | (Jahnke et al., 1989) |
| 28 | Bay of Biscay Station 1 | (Hyacinthe et al., 2001) |
| 29 | AMOR GS16-GC04 | This study and (Zhao et al., 2020) |
| 30 | AMOR GS14-GC04 | This study |

**References**

Chong, L., Berelson, W., McManus, J., and Rollins, N. (2018) Meter-scale early diagenesis of organic matter buried within deep-sea sediments beneath the Amazon River plume. *Frontiers in Marine Science* **5**: 250.

Christensen, J.P., and Rowe, G.T. (1984) Nitrification and oxygen consumption in northwest Atlantic deep-sea sediments. *Journal of Marine Research* **42**: 1099-1116.

Emerson, S., Jahnke, R., Bender, M., Froelich, P., Klinkhammer, G., Bowser, C., and Setlock, G. (1980) Early diagenesis in sediments from the Eastern Equatorial Pacific 1: pore water nutrient and carbonate results. *Earth and Planetary Science Letters* **49**: 57-80.

Engstrom, P., Penton, C.R., and Devol, A.H. (2009) Anaerobic ammonium oxidation in deep-sea sediments off the Washington margin. *Limnology and Oceanography* **54**: 1643-1652.

Hiraoka, S., Hirai, M., Matsui, Y., Makabe, A., Minegishi, H., Tsuda, M. et al. (2020) Microbial community and geochemical analyses of trans-trench sediments for understanding the roles of hadal environments. *The ISME Journal* **14**: 740-756.

Hyacinthe, C., Anschutz, P., Carbonel, P., Jouanneau, J.-M., and Jorissen, F. (2001) Early diagenetic processes in the muddy sediments of the Bay of Biscay. *Marine Geology* **177**: 111-128.

Jahnke, R.A., Emerson, S.R., and Murray, J.W. (1982) A model of oxygen reduction, denitrification, and organic matter mineralization in marine sediments. *Limnology and Oceanography* **27**: 610-623.

Jahnke, R.A., Emerson, S.R., Reimers, C.E., Schuffert, J., Ruttenberg, K., and Archer, D. (1989) Benthic recycling of biogenic debris in the eastern tropical Atlantic Ocean. *Geochimica et Cosmochimica Acta* **53**: 2947-2960.

Lund, S., Mortazavi, E., Chong, L., Platzman, E., and Berelson, W. (2018) Holocene sedimentation on the distal Amazon Fan/Demerara Abyssal Plain. *Marine Geology* **404**: 147-157.

Nunoura, T., Nishizawa, M., Hirai, M., Shimamura, S., Harnvoravongchai, P., Koide, O. et al. (2018) Microbial diversity in sediments from the bottom of the Challenger Deep, the Mariana Trench. *Microbes and Environments*: ME17194.

Thamdrup, B., Schauberger, C., Larsen, M., Trouche, B., Maignien, L., Arnaud-Haond, S. et al. (2021) Anammox bacteria drive fixed nitrogen loss in hadal trench sediments. *Proceedings of the National Academy of Sciences* **118**: e2104529118.

Zhao, R., Biddle, J.F., and Jørgensen, S.L. (2022) Introducing Candidatus Bathyanammoxibiaceae, a family of bacteria with the anammox potential present in both marine and terrestrial environments. *ISME Communications* **2**: 42.

Zhao, R., Mogollón, J.M., Abby, S.S., Schleper, C., Biddle, J.F., Roerdink, D.L. et al. (2020) Geochemical transition zone powering microbial growth in subsurface sediments. *Proceedings of the National Academy of Sciences* **117**: 32617-32626.
